# Metastatic Cecal Adenocarcinoma to the Gallbladder Presenting with Acute Cholecystitis

**DOI:** 10.1155/2018/5308585

**Published:** 2018-10-21

**Authors:** Nedal Bukhari, Marwah Abdulkader

**Affiliations:** ^1^Department of Medical Oncology, King Fahad Specialist Hospital, Dammam, Saudi Arabia; ^2^Department of Pathology, King Fahad Specialist Hospital, Dammam, Saudi Arabia

## Abstract

Colorectal cancer (CRC) is one of the most common cancers and the second highest cause of cancer-related deaths (Jemal et al., 2011). Common presentations of CRC include alterations in bowel habit, weight loss, and lower gastrointestinal bleeding. We report a case of a 74-year-old male who presented with fever and right upper quadrant pain, with positive Murphy's sign on examination. The case was initially managed with a routine cholecystectomy. Histological examination revealed a moderately differentiated adenocarcinoma with a superimposed histologically proven acute acalculous cholecystitis. CT scan done postsurgery showed a cecal mass with retroperitoneal lymphadenopathy. Biopsy result of cecal mass was remarkable for colon adenocarcinoma. We are not aware of any similar prior cases reported in English literature.

## 1. Introduction

CRC is the third most common cancer in men and the second in women worldwide [1]. Around 20% of patients will have distant metastasis at the time of initial presentation. Regional lymph nodes, liver, lungs, and peritoneum are common metastatic sites for CRC [2].

The intestinal tract venous drainage is through the portal circulation. Therefore, the first site of hematogenous spread of CRC is usually the liver, followed by the lungs, bone, and multiple other sites. However, distal rectal cancers may metastasize initially to the lungs through the inferior rectal vein, which drains into the inferior vena cava rather than into the portal venous system [3].

Throughout the literature search, we came across 2 cases of transverse colon cancers with metastasis to gallbladder masquerading as cholecystitis [4, 5].

## 2. Case Report

A 74-year-old male with history of stage III sigmoid adenocarcinoma 15 years ago treated with sigmoid colectomy followed by adjuvant 5-fluorouracil (5-FU) chemotherapy presented to his local hospital with acute worsening of epigastric pain associated with nausea and vomiting. On physical examination, the patient was febrile at 38.5°C, tachycardic, and normotensive. Abdominal examination revealed tenderness in the right upper abdomen and rigidity of the abdominal wall with positive Murphy's sign. Laboratory testing revealed a hemoglobin level of 11.5 g/dl and a white cell count of 16/*μ*l with 80% neutrophils, and other tests were within normal range (which included liver enzymes, bilirubin, LDH, lipase, and amylase).

CA19-9 was elevated at 4945 IU/ml, and the CEA level was measured at 24.11 *μ*g/l.

Abdominal ultrasound revealed a sludge and irregular thickness of the gallbladder.

**Figure 1:**
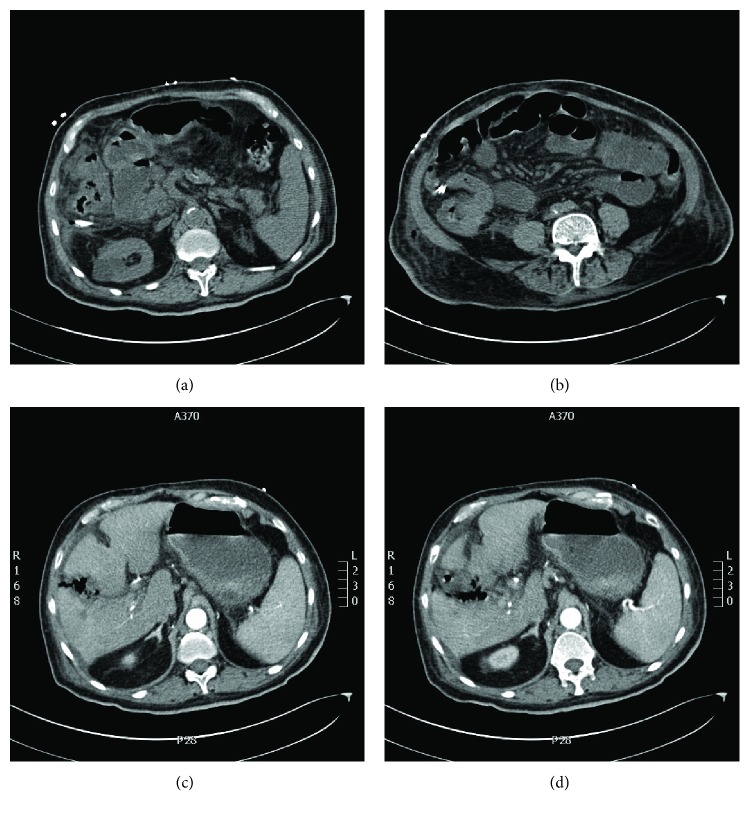
(a, b) CT Abdomen showing cecal mass. (c, d) CT abdomen showing post cholecystectomy changes.

The patient was started on intravenous broad-spectrum antibiotics immediately. Laporascopic cholecystectomy was performed the day after admission. Unfortunately, the postoperative course was complicated by a septic shock and required ICU admission for few days (Figure 1). The initial pathology of the gallbladder showed a moderately differentiated adenocarcinoma of unknown primary possibly due to gall bladder primary. Further investigations revealed a cecal mass with regional retroperitoneal lymphadenopathy.

The patient was referred to our hospital where he had a biopsy of the latter mass, and the histopathology result was consistent with a moderately differentiated adenocarcinoma of colonic origin. A comprehensive pathological review of the gallbladder specimen was performed, and reexamination and further immunohistochemical analysis including epithelial cytokeratins 7 and 20 (CK7 and CK20) and homeobox protein-2 (CDX-2) were done. Tumor cells isolated from the specimen were positive for CK20 and CDX-2 and negative for CK7.

Our patient was confirmed to have metastatic disease from colon primary; therefore, he was started on palliative capecitabine with significant symptomatic improvement reported after two cycles. He continues to tolerate chemotherapy.

## 3. Discussion

CRC is one of the most common cancers worldwide. Patients with right-side colon adenocarcinoma usually present with cachexia, weight loss, anemia, and positive fecal occult blood unlike those with left-sided colon cancers, which usually manifest with changes in bowel habit, hematochezia, and symptoms of obstruction.

The gallbladder is an extremely rare site of CRC metastasis, with very few cases reported. However, tumors like melanoma may metastasize to the gallbladder [2]. Other less common primary sites resulting in metastasis include the lung, breast, renal, and cervical malignancies [6, 7]. In a large autopsy series, metastases to the gallbladder were present in 5.8% of patients [8, 9].

Chen et al. reported a case of transverse colon cancer presenting with manifestations of cholecystitis. They suggested that invasion of the gallbladder caused an inflammatory adhesion which resulted in an acute acalculous cholecystitis [4].

Munghate et al. also described a case of transverse colon presenting with cholecystitis [5].

Adenocarcinomas are epithelial cancers arising in glandular tissues. They constitute the largest group of epithelial cancers [10].

**Figure 2:**
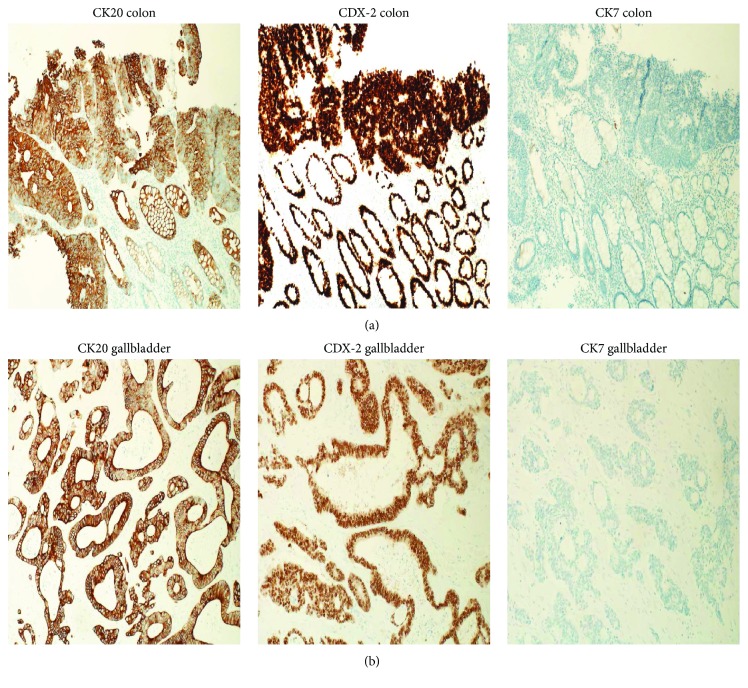
(a) Immunohistochemical analysis on the colon specimen. The specimen is CK20 positive, CK7 negative, and CDX-2 positive. (b) Final immunohistochemical analysis on the gallbladder specimen. The specimen is clearly CK20 positive, CK7 negative, and CDX-2 positive.

CK are keratin proteins found in the cytoskeleton of the epithelium (Figure 2). CK7 and CK20 expression patterns play a major role in the diagnosis of many carcinomas of epithelial etiology [11, 12]. CK7 is found in many ductal and glandular epithelial tissues, including breast, lung, ovary, and endometrium. CK20 is mainly expressed in the gastrointestinal epithelium, Merkel cells, and urothelium [11, 12]. CK20-positive/CK-negative pattern is present in the majority of intestinal adenocarcinoma and also Merkel cell carcinoma whereas the CK7-positive/CK20-negative pattern is found in breast, lung, and ovarian adenocarcinoma. Both CK7 positivity and CK20 positivity are present in gastric, pancreatic, and urothelial carcinoma [11, 12].

The homeobox protein-2 (CDX-2) test result was also helpful in our case to further distinguish colon adenocarcinoma from other gastrointestinal and hepatobiliary tumors.

CDX-2 is normally expressed within the nuclei of the intestinal epithelium, from the duodenum to the rectum, and it is a sensitive and specific marker of adenocarcinomas of intestinal origin [13, 14].

The CK7-negative/CK20-positive expression pattern with CDX2 positivity is consistent with colorectal primary, while gallbladder cancers tend to be both positive for CK7 and CK20 [12–14].

Our case had two distinguishing features; the first one was the primary site being the cecum. The second interesting finding was features of concomitant acute acalculous cholecystitis and metastatic adenocarcinoma. We hypothesize that the local spread resulted in metastasis and subsequently acute cholecystitis mimicking primary gallbladder adenocarcinoma and causing diagnostic confusion.

## 4. Conclusion

Colon adenocarcinoma metastasizing to the gallbladder is extremely rare. To our knowledge, this is the first case of primary cecal adenocarcinoma with metastasis to the gallbladder presenting with acute acalculous cholecystitis.
